# The complete chloroplast genome of *Carex laevissima* Nakai (Cyperaceae)

**DOI:** 10.1080/23802359.2022.2107455

**Published:** 2022-08-01

**Authors:** Wei Ren, Ya-ru Wang, Han-dong Zhao, Ying-zhe Wang, Zhi-feng Wang

**Affiliations:** a Jilin Academy of Agricultural Sciences, Changchun, China; b School of Life Science, Jilin Normal University, Siping, China; c Changchun Agricultural Expo Garden, Changchun, China

**Keywords:** *Carex*, chloroplast, genome assembly, phylogenetic analysis

## Abstract

*Carex laevissima* Nakai 1914 (Cyperaceae) is vital for ecological conservation and land virescence, and has high ornamental value. Here the chloroplast genome of *Carex laevissima* was assembled and systematically analyzed for further genetic research of *Carex* plants. The chloroplast sequence of *Carex laevissima* was 188,029 bp in length, including two inverted repeat (IR) regions of 36,699 bp each, a large single-copy (LSC) region of 106,171 bp and a small single-copy region (SSC) of 8460 bp. The overall GC content is 34.0%. It contains 133 genes, including 89 protein-coding, 36 tRNA, and eight rRNA genes. Phylogenetic analysis showed that *Carex laevissima* is most closely related to *Carex neurocarpa*.

The genus *Carex* is important because of its sand stabilization, ornamental value, medicinal properties and animal feeding, and belongs to the Cyperaceae family. It is widely distributed in China, New Zealand, Germany and North America, with more than 2000 species around the world. The genetic characteristics of 79 *Carex* germplasms have been investigated using SSR markers (Liu et al. 2021). However, there was no mention of *Carex laevissima* Nakai 1914 being widely distributed in Northeast China, which has deeper roots, more tillers, and stronger cold and drought resistance. Chloroplast genomes are widely used for germplasm identification, genetic studies, phylogenetic analysis, and evolutionary relationships. In this study, chloroplast genome sequence of *C. laevissima* is first reported. In addition, the phylogenetic analysis is useful for further genetic diversity analysis and scientific research on *Carex* plants.

Young fresh leaves of *C. laevissima* were collected from Baicheng City, Jilin Province, China (45°49′4.3″N, 123°9′1.0″E). This study complied with National Wild Plant Protective Regulations and we were allowed by the Jilin Academy of Agricultural Sciences to collect the required samples of plant material. A specimen was deposited at the Institute of Agricultural Biotechnology, Jilin Academy of Agricultural Sciences (contact Wei Ren, renwei@cjaas.com) under voucher number JAAS-TC-17-5. Total genomic DNA was extracted using a modified CTAB method. The libraries were constructed with an average length of 350 bp using the Next era XT DNA Library Preparation Kit (Illumina, San Diego, CA). Library sequencing was performed by Huitong-Biotechnology (Shenzhen, China) using the Illumina NovaSeq 6000 platform. Raw sequence reads were edited using the NGS QC Tool kit (Patel and Jain 2012). A total of 4.59 Gb clean data were de novo assembled by SPAdesv.3.11.0 software (Bankevich, et al. 2012). Then, the assembled chloroplast genome was annotated via PGA using the chloroplast genome of *Carex agglomerate* C. B. Clarke 1903(MT795185) as the reference. Finally, the complete chloroplast genome sequences and annotations of *C. laevissima* were submitted to GenBank under accession number MZ846224.

The length of the *C. laevissima* chloroplast genome sequence is 188,029 bp with a typical quadripartite structure. It contains two inverted repeat (IR) regions of 36,699 bp each, which are separated by a large single-copy (LSC) region of 106,171 bp and a small single-copy (SSC) region of 8460 bp. The overall GC content is 34.0%. A total of 133 genes were predicted, including 89 protein-coding genes (PCGs), 36 tRNA genes, and eight rRNA genes. In addition, fifteen genes (atpF, ndhA, ndhB, petB, petD, rpl2, rpl16, rps16, rpoC1, trnA, trnG, trnI, trnK, trnL, and trnV) contained one intron, and clpP and ycf3 had two introns. Trans splicing existed in the rps12 gene. Compared with the chloroplast genome of *C. agglomerata* (184,157 bp, MT795185), the chloroplast genome of *C. laevissima* reported here has much longer IR and shorter SSC regions (Xun, et al. 2021). Mohanta et al. （2020）reported that approximately 10.31% of the plant chloroplast genomes had lost the inverted repeats (IR), and C. *laevissima* contained two inverted repeat (IR) regions. *PsaM, Psb30, ChlB, ChlL, ChlN*, and *Rpl21* were not found in C. *laevissima* chloroplast genome, but The *Rpl20* gene was existed, which was in line with their research.

To analyze the C. *laevissima* chloroplast genome, the complete chloroplast genomes of 32 plant species, including 31 related members (8 genera) and one outgroup taxon (*Cryptanthus acaulis*, Bromeliaceae, NC_061333.1) were used to identify their phylogenetic position. We aligned the 65 homologous protein-coding genes in each of the 32 complete chloroplast genomes using the MAFFT 7.037 software (Katoh and Standley 2013). Then, the maximum-likelihood (ML) tree was constructed via IQ-TREE v1.6.12 (Nguyen, et al. 2015) under the HIVb + F+R3 model with 1000 bootstrap replicates. The results showed that *C. laevissima* was in a clade with *Carex neurocarpa*, *Carex gibba* and *Carex kokanica*. Meanwhile, *C. laevissima* was most closely related to *C. neurocarpa*. Chloroplasts, as the semiautonomous organelles, are vital for plant cell metabolism (Yu et al. 2014) and photosynthesis (Stern et al. 1997). These data are beneficial for future research on chloroplast genome evolutionary relationships and variety breeding in *Carex* plants (Figure 1).

**Figure 1.**
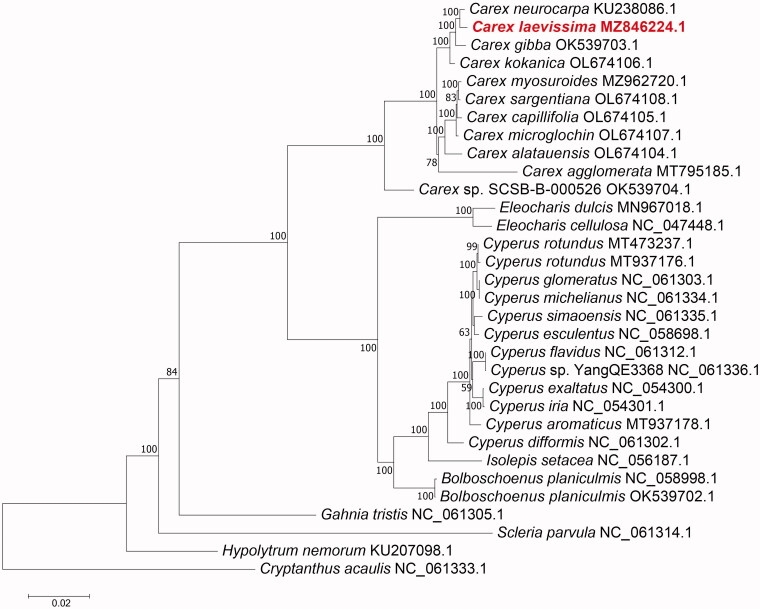
The maximum likelihood phylogenetic tree is based on 65 homologous protein-coding genes in 32 species. Note: Numbers to the right of the nodes represent the bootstrap value for 1000 replicates.

## Author contributions

Zhi-feng Wang designed the experiments. Wei Ren prepared the sample, completed the drafting of the paper. Ya-ru Wang performed the experiments. Han-dong Zhao analyzed the data. Ying-zhe Wang performed the software, data acquisition and revised the manuscript. All authors approve the final version to be published and agree to be accountable for all aspects of the work.

## Data Availability

The genome sequence data that obtained at this study are openly available in GenBank of NCBI (https://www.ncbi.nlm.nih.gov/) under the accession number of MZ846224.The associated BioProject, Bio-Sample and SRA numbers are PRJNA755795, SAMN20841307 and SRR15506181, respectively.
